# PDXliver: a database of liver cancer patient derived xenograft mouse models

**DOI:** 10.1186/s12885-018-4459-6

**Published:** 2018-05-09

**Authors:** Sheng He, Bo Hu, Chao Li, Ping Lin, Wei-Guo Tang, Yun-Fan Sun, Fang-You-Min Feng, Wei Guo, Jia Li, Yang Xu, Qian-Lan Yao, Xin Zhang, Shuang-Jian Qiu, Jian Zhou, Jia Fan, Yi-Xue Li, Hong Li, Xin-Rong Yang

**Affiliations:** 1 School of Life Science and Technology, ShanghaiTech University, Shanghai, 201210 China; 2 Department of Liver Surgery and Transplantation, Liver Cancer Institute, Zhongshan Hospital, Fudan University; Key Laboratory of Carcinogenesis and Cancer Invasion of Ministry of Education, Shanghai, 200032 China; 3 CAS Key Laboratory for Computational Biology, CAS-MPG Partner Institute for Computing Biology, Shanghai Institute for Biological Sciences, Chinese Academy of Sciences, Shanghai, 200031 China; 4 University of the Chinese Academy of Sciences, Beijing, 100049 China; 5 School of Life Sciences and Biotechnology, Shanghai Jiao Tong University, Shanghai, 200031 China

**Keywords:** Liver cancer, PDX model, Drug response, Database

## Abstract

**Background:**

Liver cancer is the second leading cause of cancer-related deaths and characterized by heterogeneity and drug resistance. Patient-derived xenograft (PDX) models have been widely used in cancer research because they reproduce the characteristics of original tumors. However, the current studies of liver cancer PDX mice are scattered and the number of available PDX models are too small to represent the heterogeneity of liver cancer patients. To improve this situation and to complement available PDX models related resources, here we constructed a comprehensive database, PDXliver, to integrate and analyze liver cancer PDX models.

**Description:**

Currently, PDXliver contains 116 PDX models from Chinese liver cancer patients, 51 of them were established by the in-house PDX platform and others were curated from the public literatures. These models are annotated with complete information, including clinical characteristics of patients, genome-wide expression profiles, germline variations, somatic mutations and copy number alterations. Analysis of expression subtypes and mutated genes show that PDXliver represents the diversity of human patients. Another feature of PDXliver is storing drug response data of PDX mice, which makes it possible to explore the association between molecular profiles and drug sensitivity. All data can be accessed via the Browse and Search pages. Additionally, two tools are provided to interactively visualize the omics data of selected PDXs or to compare two groups of PDXs.

**Conclusion:**

As far as we known, PDXliver is the first public database of liver cancer PDX models. We hope that this comprehensive resource will accelerate the utility of PDX models and facilitate liver cancer research. The PDXliver database is freely available online at: http://www.picb.ac.cn/PDXliver/

## Background

Patient-derived xenograft (PDX) models are generated by directly transplanting cancer patients’ tumor samples to immune-compromised mice. It has been widely applied to multiple cancer types, such as breast cancer, lung cancer and colon cancer [1–4]. PDX models recapitulated the histologic, genomic, expression and biological characteristics of the corresponding primary tumors [5–8]. Additionally, the response of PDX models to drug treatment are remarkable correlated with clinical outcome, which made it possible to establish personalized xenograft model to help make therapeutic decision and guide the cancer treatment [9–12]. For example, Manuel Hidalgo et al. generated 14 PDX models for 14 patients and treated tumorgrafts with 63 different drugs; an effective drug in the PDX model was identified for 12 patients [13]. Hui Gao et al. have established ~ 1000 PDX models involving six cancer types, used these models to in vivo screen drug and compounds, and successfully reveal some resistance mechanisms [14]. Therefore, PDX models are thought to be the best pre-clinical models for translational drug development in cancer research.

Liver cancer is the second most common causes of cancer-related deaths with an estimated nearly, 29,200 new cases and 40,710 fatalities occurred United States in 2017 [15], with the highest incidence rates reported in China. For patients with unresectable liver cancers, sorafenib is the only FDA approved standard first-line drug. However, sorafenib only improved the overall survival by nearly 3 months [16, 17]. The current genome sequencing revealed that 28% of liver cancer patients harbored at least one targetable mutation by FDA-approved drugs [18]. In future, these patients may benefit from targeted treatment if the effectiveness of drug is further validated by PDX models.

The first liver cancer PDX model was reported in 1996, but this field progressed slowly due to the very low engraftment rate [19–23]. In the last five years, the engraftment rate was increased to nearly 40% by improved experimental methods and many liver cancer PDX models had been generated. A cohort of 65 stable liver cancer PDX models were established and revealed that lenvatinib, a FGFR1 inhibitor, presentated better therapeutic effect than sorafenib in the models expressing high levels of FGFR1 gene [24]. Although PDX models have achieved useful results in liver cancer treatment, there still exist some problems, such as engraftment failure for 59% patients, long-time (2~ 4 months) for the tumor to engraft. With more cumulated PDX models, it will be feasible to predict the drug response of new patients. Such algorithms have been used in large-scale cancer cell line studies. Machine learning or regression models were built to predict drug sensitivity based on molecular profiles, including gene expression, somatic mutations and copy-number alterations [25, 26]. Nevertheless the current studies of liver cancer PDXs are scattered in different literatures. An integrated database with unified format will facilitate the application of liver cancer PDXs.

Here we built a database PDXliver, which collected liver cancer PDX models and provided comprehensive genome, transcriptome and drug response data. Currently PDXliver contains 116 PDX mouse models from 116 Chinese liver cancer patients, 26 of which have drug response data. Sixty-five models were curated from public papers and others came from in-house PDX platform. We also developed tools to help users to explore the omics data of PDX models. This new resource will facilitate the drug sensitivity prediction and precision medicine of liver cancer.

## Construction and content

### Implementation

**Fig. 1:**
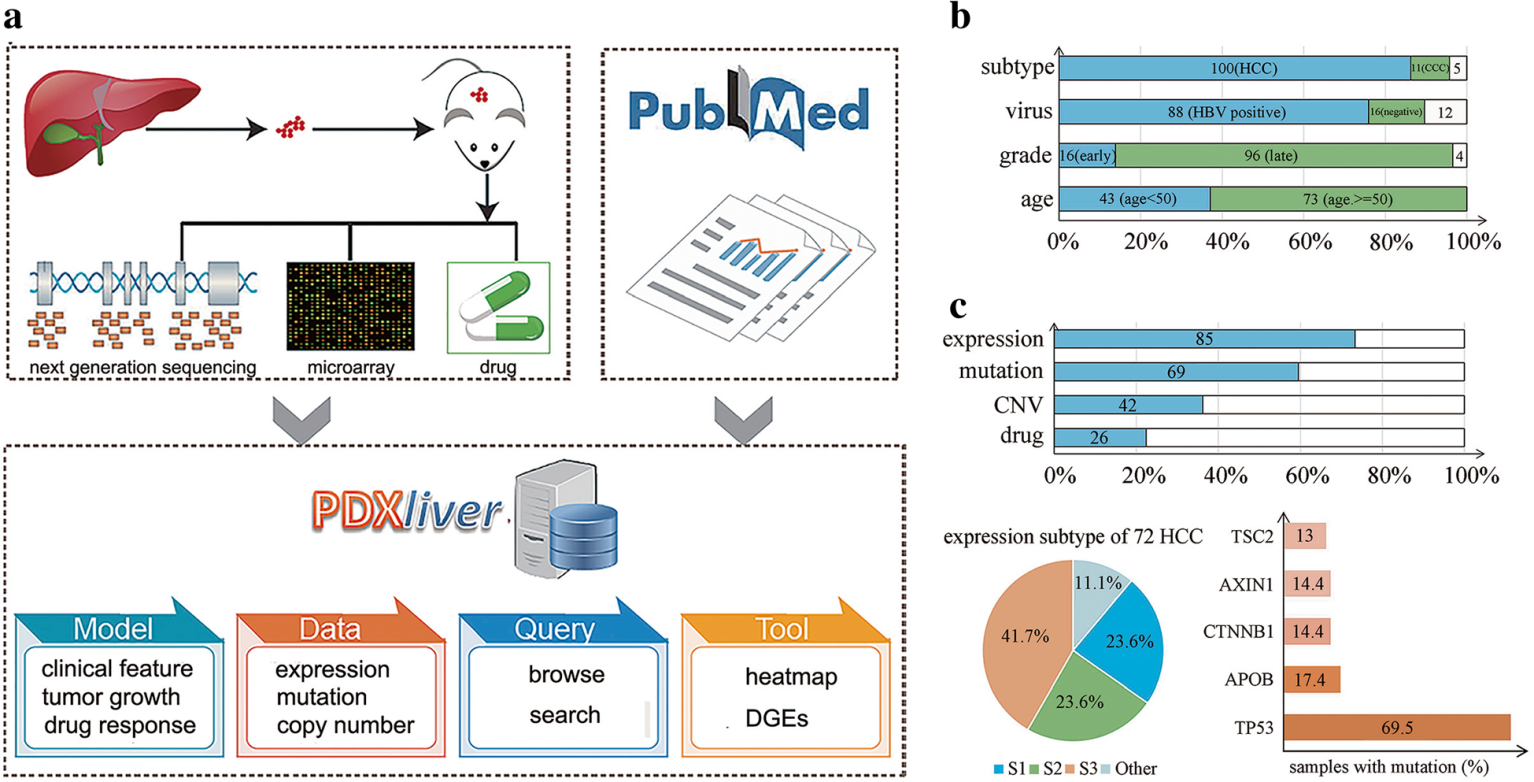
Overview of PDXliver database. **a** Workflow of collecting PDX models and designing PDXliver database. PDXliver contains multi-omics data from both the in-house experimental platform and published literatures. **b** Statistics of clinical indicators. **c** Number of PDX models which have gene expression, somatic mutation, copy number alteration (CNA) and drug treatment data. HCC were classified into three expression subtypes based on a previous published nearest template prediction method. Genes with frequent somatic mutations (> 10%) were shown in a barplot

We integrated PDX models from the in-house PDX experimental platform and public literatures [24]. Models that came from the same batch or literature were called a dataset. To facilitate the management of massive PDX mouse models, we also set naming rules for patients and xenografts. Each patient was randomly assigned an identifier (e.g.: PD0001). Mice from this patient were named using the combination of patient’s identifier and the passage number. F1 is the 1st-passage xenograft, F2 is the 2nd-passage, and so on. Clinical, molecular and drug sensitivity data were stored into database if they were available. Three main function blocks (search, browse, tools) were developed to display the characteristics of PDX models (Fig. 1a).

The PDXliver database was built in RedHat Linux server (4.4.7–11) and the web interface was implemented using a combination of JavaScript and PHP scripts. All the data were stored in a MySQL (version 5.7.9) database. Data processing and visualization were performed by R language or JavaScript plugin. PDXliver can be accessed via the major browsers such as Chrome, Safari, Edge, and Firefox.

### Establishment of PDX models

4–5 weeks old male non-obese diabetic severe combined immunodeficiency (NOD/SCID) mice as transplant recipient models were raised in the aseptic environment. Liver cancer tumor samples were cut into pieces within 1 h after removal from patients. Tissue fragments were incubated in DMEM medium supplemented with 50% Matrigel™ (BD; 356,234), 10 ng/mL epidermal growth factor (Gibco; PHG0314), 10 ng/ml basic fibroblast growth factor (Gibco; PHG0264), 100 U/mL penicillin, and 100 U/mL streptomycin for 30 min. Tumor tissues with the incubation mix (Matrigel plus growth factors) were transplanted into the right flanks of mice (*n* = 3; 4–5 weeks old, Shanghai Institute of Material Medicine, Chinese Academy of Science) subcutaneously with a No. 20 trocar. Animal care and experimental protocols were approved by the Shanghai Medical Experimental Animal Care Commission. The tumor growth was recorded three times a week from the measurement of length (L)and width (W) with caliper and calculated as tumor volume (TV, mm3) = 0.5 × L × W2 (mm). To explore the intrinsic sensitivity of the first-line drug of treatment for liver cancer, some PDX models were randomly selected for drug experiments. To estimate the performance of drugs, a criterion named Tumor Growth Inhibition (TGI) was used. Tumor Growth Inhibition is equal to the ratio of the experimental group tumor volume and the control group tumor volume. When the tumor in the control group mice grew to the definite dimension, both the control and experimental group mice were killed and fresh tumor samples were obtained to do microarray or next generation sequencing. All clinical data was anonymized to protect patient privacy.

### Collection of public PDX models

To expand the capacity of our database, we collected clinical and molecular information of PDX models from literatures. PubMed database was searched using keywords “liver cancer” or “hepatocellular carcinoma”, and “PDX” or “patient-derived xenograft”. It returned 148 records by Feb 10, 2018. After the manual check, 52 literatures established liver cancer PDX mice from patients, but only one of them provided both clinical information and high-throughput molecular data [24]. If the original authors had uploaded their experimental data to GEO or other databases, we downloaded the raw data and processed it into a unified format (detailed methods were described in the next paragraph). If raw data was not available but there was processed data in supplementary material, we directly stored the processed data into database.

### Data processing

The raw data was processed into unified format before storing into database.

1. Affymetrix genome-wide human SNP 6.0 array

Raw data were stored in CEL files. Genotypes were called by the CRLMM algorithm in R package “oligo”. SNP identifiers were mapped to dbSNP ID and gene symbols by R package “pd.genomewidesnp.6”.

2) Exome sequencing

Raw data were stored in FASTQ files. Low-quality reads were filtered, and then the remained reads were mapped to human genome (hg19) by BWA algorithm. Duplicated reads were removed by the picard tool. SNVs and indels were called by GATK based on the GATK best practices. Somatic mutations were identified by VarScan2 if the paired tumor and control samples were available. The potential functional effects of mutations were annotated by ANNOVAR. We collected 72 significantly mutated genes from 6 published genomes studies of liver cancer patients. Functional mutations (non-synonymous, splicing, stop-gain and stop-loss SNVs; exonic indels) in these significantly mutated genes were stored into database.

3) Affymetrix gene expression array

Raw data were stored in CEL files. Expressions of probes were estimated by the Robust Multichip Average algorithm in R package “affy”, and normalized by quantile normalization. For genes with multiple probes, the mean of all probes was used as gene expression. The gene expression genes were log2 transformed and then stored into database.

4) RNA sequencing

Raw data were stored in FASTQ files. Low-quality reads were filtered using NGSQC Toolkit. Then high-quality reads were mapped to human genome (hg19). Gene expression levels were estimated by the RSEM algorithm, and normalized by the trimmed mean of M-values *(TMM*) normalization method.

For most of the in-house DPX models, raw data have been submitted to public repositories (GEO). PDXliver webpages provide links to the public repositories.

### Data statistics

The PDXliver database was designed to provide a data storage, search, and analysis system for liver cancer mouse xenografts (Fig. 1a). Currently, it contained three datasets. One dataset were obtained from a public literature [24]. Another two datasets came from our in-house PDX experimental platform (the Liver Cancer Institute of ZhongShan Hospital, Fudan University, Shanghai, China); some PDX models are firstly publicly available in PDXliver. Table 1 gives the source of each dataset, the number of PDX models with molecular profiles or drug treatment. A total of 116 patients have stable PDX models, some patients have multiple serially passaged xenografts. All patients have been comprehensively annotated with clinical information, such as age, gender, virus infection and tumor stage (Table 2, Fig. 1b). Since all patients are Chinese, most of them are HBV positive (*n* = 88). Hepatocellular carcinoma (HCC) is the major histopathologic subtype (*n* = 100), followed by cholangiocarcinoma (*n* = 11). A part of models have genome-wide expression profiles (*n* = 88), germline variations (*n* = 40), somatic mutations (*n* = 69) and copy number alterations (*n* = 42). Expression profiles of 72 HCC were available and they were classified into three subgroups using a previous public method [27]: S1 (23.6%), S2 (23.6%) and S3 (41.7%) (Fig. 1c). TP53 (69.5%) is the most frequently mutated gene in liver cancer PDX models; its frequency is higher than the reported frequency (25%~ 35%) in liver cancer patients [28, 29]. Mutation frequency of another four genes (APOB, CTNNB1, AXIN1, TSC2) are higher than 10%. We also provide histological staining (*n* = 40), tumor growth curve (*n* = 40), and drug response data (*n* = 26) for the in-house PDX models.

**Table 1:** Data source and statistics of PDXliver database. Multiple PDX models from the same patient were counted only once

Data Set	Patient	Transcriptome		Genome		Drug treatment	Source	Reference
		patient	platform	patient	platform			
DataSet1	46	40	Affymetrix Human Genome U133 Plus 2.0 Array (GPL570)	40	Affymetrix Genome-Wide Human SNP 6.0 Array	21	ZhongShan Hospital	unpublished
				13	Exome sequencing			
DataSet2	65	43	Affymetrix Human Gene Expression Array (GPL15207)	42	Affymetrix Genome-Wide Human SNP 6.0 Array	0	WuXi AppTech	[24]
				56	Exome sequencing			
DataSet3	5	5	RNA sequencing	/		5	ZhongShan Hospital	unpublished

**Table 2:** Clinical information of 116 liver cancer patients

Clinical indexes		No. of Patients
Age (y)	< 50	43
	≥50	73
Gender	Female	20
	Male	96
Tumor differentiation	Early stage (I-II)	16
	Late stage (III-IV)	96
HBV	Positive	88
	Negative	16
HCV	Positive	1
	Negative	47
Tumor encapsulate	Complete	30
	None	20
Tumor subtype	Hepatocellular carcinoma	100
	Cholangiocarcinoma	11
	Other	5

## Utility and discussion

### Data browse and search

**Fig. 2:**
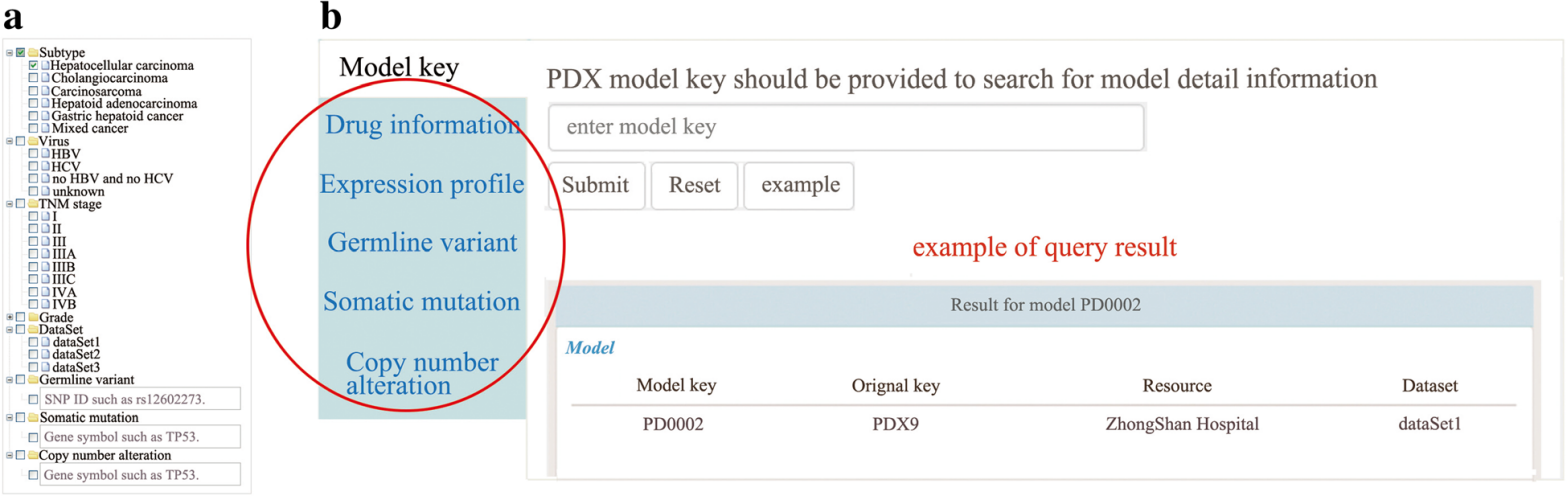
Screenshots of the “Browse” and “Search” pages. **a** “Browse” page. PDX models can be browsed by histopathologic subtype, tumor grade, virus infection state, and data sources. **b** “Search” page. PDX models can be queried by the model identifier and drug name. Gene expression, germline variants, somatic mutation and copy number alterations can be retrieved by gene symbol (as shown in the red oval). An example of corresponding result was showed in the lower right

To help users quickly understand and query PDXliver, we designed the “Browse” and “Search” pages (Fig. 2).

The “Browse” page allows users to query PDX models by histopathologic subtype, tumor grade, virus infection and data source (Fig. 2a). Users can select the combination of multiple conditions. Results will be returned in a table in which each row represents a model including some summary information. Meanwhile, users can go to the detailed page of PDX model by clicking the hyperlink.

**Fig. 3:**
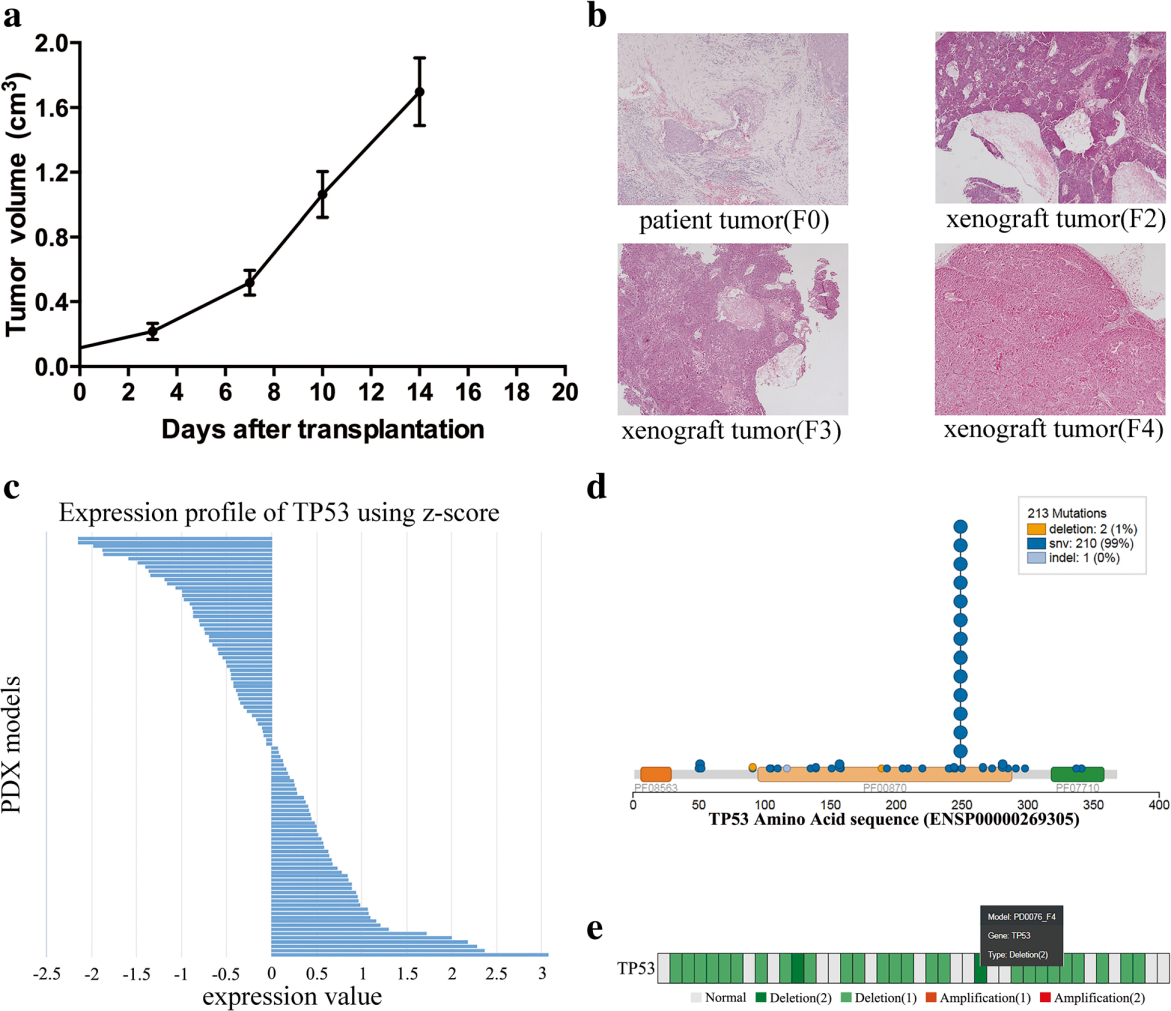
The detailed page of PDX model or gene. **a** Growth curve of the transplanted tumors. **b** Pathological images of patient tumors (F0) and mouse xegnografts (between F2 and F4) from patient PD0003. **c** Expression profile of TP53 gene. Expression values were normalized by the z-score. **d** Somatic mutations in TP53, including mutation types and locations. **e** Copy number alternations of TP53. This graph only shows PDX models whose CNA data are available. Gray and colored boxes indicate normal and altered copy number, respectively

The “Search” page contains six query frames (Fig. 2b). Firstly, users can exactly find the PDX mouse model if they know its identifier (e.g. PD0001). It will link to the detailed page of PDX model, including clinical characteristics of the associated patient, engraftment conditions, drug treatment, histopathology image and tumor growth curve (Fig. 3a). Some entries may be empty if the data is unavailable. A part of transplantable xenograft lines have been stably maintained over multiple passages, e.g. F1- to F4- generations of PD0003. Our previous work have proved that the histological, genomic and transcriptomic characteristics of PDX mice were remarkable stable across sequential passages. The result page also showed the similarity of histopathology images among serially transplanted xenografts (Fig. 3b). Secondly, users can retrieve PDX mice that have been treated by the given drug. After querying a drug name, a result table will be returned to display the model identifier, drug dose, frequency and duration of drug treatment, and drug response rate (TGI). Thirdly, user can obtain the expression profile of one gene by querying its gene symbol. Expression values in all PDX models are visualized by a barplot (Fig. 3c). Fourthly, users can query a single nucleotide polymorphism (SNP) site by its dbSNP ID. The result page contains the basic information of SNP, such as location and associated gene; it also shows its genotypes in different PDX models. Fifthly, users can browse somatic mutations of a given gene in liver cancer PDXs. The result page shows chromosome position, reference and alternative alleles, mutation type, amino acid change, and potential function effect (Fig. 3d). The last query item allows users to search the copy number alterations with gene symbol. It will return the detail copy number variations in PDX models if the associated data are available (Fig. 3e).

### Analysis tools

#### Visualization of molecular profiles

**Fig. 4:**
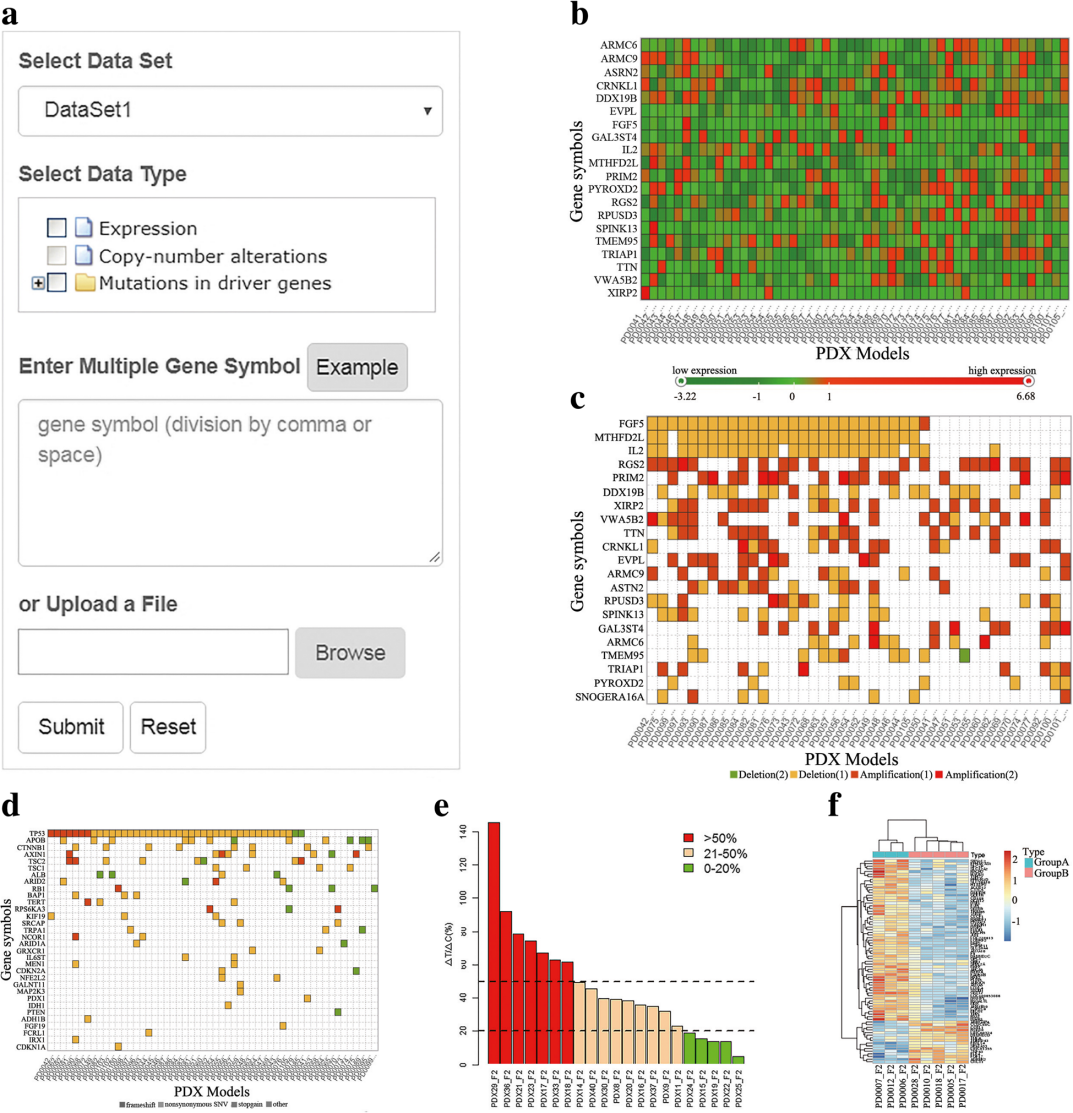
Screenshots of the analysis tools. **a** Input parameters of the “heatmap” tool. **b** Expression heatmap of the example genes in dataset1. Red indicates high expression and green means low expression. **c** Example heatmap of the somatic copy-number alterations in dataset2. Colors represent the type and number of copy number alterations. **d** Heatmap of the somatic mutations for 45 significantly mutated genes in dataset2. Colors represent the mutation types, such as frameshift indels, non-synonymous, splicing, stop-gain and stop-loss SNVs. **e** Response of 16 Hepatocellular carcinoma PDXs to Sorafenib. According to the criteria of the Division of Cancer Treatment (NCI), we defined a response as 0–20% TGI; stability as 21–50% TGI; and tumor progression as > 50% TGI. **f** Example result of the “differential expression analysis” tool. Genes were differentially expressed in sorafenib-sensitive and resistant groups (T-test, *P* < 0.01, FoldChange> 2), which may be related to the response of sorafenib

The “HeatMap” tool allows users to intuitively visualize the molecular profiles of PDX models. To draw a customizable heatmap, users need to select a dataset and a molecular type, and provide a list of genes (Fig. 4a). There are three alternative molecular types: gene expression, somatic mutation and somatic copy number alteration (SCNA). The available molecular type may be different based on the source of datasets. For example, dataset1 has gene expression and somatic mutation data but does not have SCNA; therefore SCNA is not an optional molecular type after selecting dataset1. For somatic mutation, the heatmap only plots the significantly mutated genes in liver cancer patients [30]. For gene expression and SCNA, users need to provide a list of genes by entering gene symbols in the text area or uploading a file that contains gene symbols. The gene symbols should be separated by comma or space.

Once users input these necessary parameters, a customized heatmap will be plotted. Figure 4b shows an example of expression heatmap. Each row is a gene and each column is a PDX mouse model. Gene expression values are converted to Z-score. Color represents the Z-score of gene expression values. Red indicates high expression and green means low expression. In a heatmap of copy-number alterations, the variation-type (amplification or deletion) and the number of copy number variations are respectively painted with different colors (Fig. 4c). The heatmap of somatic mutation is similar, with color reflected different types of somatic mutation, such as frame-shift indels and non-synonymous SNVs (Fig. 4d).

#### Differential expression analysis

The purpose of this tool is to identify genes whose expressions are significantly different between two groups of PDX models. Expression profiles from different datasets may have batch effect; pathological subtypes of liver cancer may display different expression patterns. Therefore, we provided two parameters (dataset and histological type) to make the selected PDX models more comparable. Then users can classify PDX models into two groups by their clinical indicators, such as “response to sorafenib” and “status of HBV infection”. All genes were tested by the given statistical method. Significant genes were selected out by the user defined *P*-value and fold change.

To illustrate the use of this tool, we tried to find genes associated sorafenib response by comparing sensitive PDXs and resistant PDXs. We selected “dataset1”, “Hepatocellular carcinoma” and “Drug response to sorafenib” in the first three steps. Then the webpage showed 16 PDX mice whose responses to sorafenib were evaluated by tumor growth inhibition (Fig. 4e). According to the criteria of the Division of Cancer Treatment (NCI), PDXs whose TGI < 20% were defined as having response and PDXs whose TGI > 50% were defined as tumor progression. Therefore, we selected 3 sensitive PDXs and 5 resistant PDXs in the step 4. We compared the sensitive group and resistant group by the two-tailed T-test. There were 77 significantly differential genes between two groups (Fig. 4f) (*P* < 0.01, FoldChange> 2), indicating potential association with sorafenib response. We also realized that the statistical power might be low due to the small number of PDX models, more PDXs are needed to make the results more reliable. Our tool will make it easier to get the analysis results of new data.

## Conclusion

As a preclinical model, PDX models represent the original features of patient tumors in many ways and have been shown to be predictable for the drug response of patients. Nevertheless, the progress of PDX models is relatively slower in liver cancer than it in other cancer types, such as breast cancer and lung cancer. It is important to establish a comprehensive PDX cohort that represents the diversity of the human liver cancer patients. The PDXliver is designed to provide a bioinformatic platform to collect and analysis liver cancer PDXs data. In recent years, genomic and transcriptomic studies of human liver cancer patients have made great contributions. The genomic studies revealed mutation landscape of liver cancer, promoting the understanding of cancerogenesis. The transcriptomic studies classified liver cancer into different expression subtypes, promoting the understanding of cancer heterogeneity. However, these studies lack of the information of treatment response. It is difficult to directly associate gene with drug through patients. PDXliver stores hundreds of liver cancer PDX models, which covers the significantly mutated genes and expression subtypes of liver cancer patients. Therefore, PDXliver retains the heterogeneity of human liver cancers. It will be valuable for precision medicine.

To facilitate the utility of liver cancer PDX mice models, PDXliver has several features. Firstly, PDXliver has the largest number of liver cancer PDX models so far and the number will continue to increase in future. Secondly, PDXliver categorizes all clinical data, genomic data, transcriptome data and drug responses data for easy search and access, which makes users quickly gain insights into the models. Thirdly, the analysis tool allows users to compare the molecular profiles among multiple PDX models. We expect that PDXliver will become a very useful resource for liver cancer PDXs research.

As far as we known, PDXliver is the first public database for liver cancer PDX mice models. We searched PubMed and did not find other databases specifically designed for PDX mouse models. One similar database, Mouse Tumor Biology Database (MTB http://tumor.informatics.jax.org/mtbwi/index.do), provides information on diverse mouse models for human cancers. MTB contains 440 PDX models from the Jackson laboratory, but only 2 models originated from liver (hepatoid adenocarcinoma). Another software cBioPortal provides a public framework for storing and visulizing cancer genomic data, which generated from tissue biopsy samples of cancer patients. However, PDXliver focuses on the liver cancer patient-derived xenograft models; the combination of drug sensitivity and omics data will be more useful for pharmacogenomics research.

Although PDXliver is the most comprehensively resources for liver cancer PDX models, there are still limitations. Expression profiles in different datasets were generated from different experiment platforms or different analysis methods, which may be not comparable. Furthermore, the public dataset didn’t have drug response data. Even for the in-house PDX models, the major drug is sorafenib and only a few models have drug sensitivity values for multiple drugs. We plan to treat PDX models with more drugs in future. An ongoing project in our lab has constructed another 50 liver cancer PDX models with drug response. These models and future new models will be stored into PDXliver. More models with available drug response data will make this database much more useful for drug sensitivity prediction. In addition, some important information such as therapeutic effects and survival time of patients are not available due to the short follow-up time, we will add these data in future to enrich the database contents.

## Abbreviations

GEO: Gene Expression Omnibus
HCC: Hepatocellular carcinoma
MTB: Mouse tumor biology database
NOD/SCID: Non-obese diabetic severe combined immunodeficiency
PDX: Patient-derived xenograft
TGI: Tumor growth inhibition
